# Coenzyme Q10 and Oxidative Stress: Inflammation Status in Hepatocellular Carcinoma Patients after Surgery

**DOI:** 10.3390/nu9010029

**Published:** 2017-01-04

**Authors:** Hsiao-Tien Liu, Shao-Bin Cheng, Yi-Chia Huang, Yin-Tzu Huang, Ping-Ting Lin

**Affiliations:** 1 Division of General Surgery, Department of Surgery, Taichung Veterans General Hospital, Taichung 40705, Taiwan; langhsky@vghtc.gov.tw (H.-T.L.); sbc@vghtc.gov.tw (S.-B.C.); 2 Department of Nutrition, Chung Shan Medical University, Taichung 40201, Taiwan; ych@csmu.edu.tw (Y.-C.H.); scorpio5220@hotmail.com (Y.-T.H.); 3 School of Medicine, Chung Shan Medical University, Taichung 40201, Taiwan; 4 Department of Nutrition, Chung Shan Medical University Hospital, Taichung 40201, Taiwan

**Keywords:** coenzyme Q10, oxidative stress, inflammation, hepatocellular carcinoma, surgery

## Abstract

(1) Background: Hepatocellular carcinoma (HCC) is the second leading cause of cancer deaths worldwide, and surgical resection is the main treatment for HCC. To date, no published study has examined the status of coenzyme Q10 in patients with HCC after surgery. Thus, the purpose of this study was to investigate the correlations between the level of coenzyme Q10, oxidative stress, and inflammation in patients with HCC after surgery; (2) Methods: 71 primary HCC patients were recruited. Levels of coenzyme Q10, vitamin E, oxidative stress (malondialdehyde), antioxidant enzymes activity (superoxidase dismutase, catalase, and glutathione peroxidase), and inflammatory markers (high sensitivity C-reactive protein; tumor necrosis factor-α; and interleukin-6) were measured; (3) Results: Patients with HCC had a significantly lower levels of coenzyme Q10 (*p* = 0.01) and oxidative stress (*p* < 0.01), and significantly higher levels of antioxidant enzymes activities and inflammation after surgery (*p* < 0.05). The level of coenzyme Q10 was significantly positively correlated with antioxidant capacity (vitamin E and glutathione peroxidase activity) and negatively correlated with inflammation markers after surgery; (4) Conclusion: Hepatocarcinogenesis is associated with oxidative stress, and coenzyme Q10 may be considered an antioxidant therapy for patients with HCC, particularly those with higher inflammation after surgery.

## 1. Introduction

The most recent reports from the World Health Organization (WHO, 2014) and the Ministry of Health and Welfare (2014) in Taiwan indicated that hepatocellular carcinoma (HCC) is the second leading cause of cancer deaths [1,2]. Surgical resection is the main treatment for primary HCC [3]. During or after surgical procedures, there is a physiological stress response that involves activation of inflammatory, endocrine, metabolic, and immunological mediators [4]. Oxidative stress, which is defined as a disturbance in the balance between the productions of reactive oxygen species (ROS) and antioxidant defenses [5]. The increase in the oxidative stress during or after surgery may be associated with increasing complications such as myocardial injury, sepsis, pulmonary edema, kidney and liver failure, and increased mortality [4,6,7]. As a result, oxidative stress and inflammation status is related to the prognosis of the disease after surgery in HCC patients.

Hepatocytic proteins, lipids, and DNA may affect ROS that are primarily produced in the mitochondria [8]. Coenzyme Q10 (also called ubiquinone) is a lipid-soluble benzoquinone that has 10 isoprenyl units in its side chain and is a key component of the mitochondrial respiratory chain for adenosine triphosphate synthesis [9,10]. Studies has been indicated that coenzyme Q10 is an intracellular antioxidant can protects membrane phospholipids, mitochondrial membrane protein, and low density lipoprotein-cholesterol (LDL-C) from free radical-induced oxidative damage [11,12]. In vitro or in vivo studies have demonstrated that coenzyme Q10 not only plays an antioxidant, but also has anti-inflammation effects [13,14] by modulating the expression of cyclooxygenase-2 and nuclear factor-κB (NF-κB) in the liver tissue of rats with HCC [15,16]. However, to date, no published study has examined the status of coenzyme Q10 in patients with HCC and the correlation between oxidative stress and inflammation with coenzyme Q10 after surgery. Thus, the purpose of this clinical study was to investigate the levels of coenzyme Q10, oxidative stress, and inflammation status in patients with HCC before and after surgery.

## 2. Materials and Methods

### 2.1. Participants 

A total of 71 patients were diagnosed with primary HCC (International Classification of Diseases 9, code 155.0) were recruited from the Division of General Surgery of Taichung Veterans General Hospital, which is a teaching hospital in Taiwan. We excluded patients who were younger than 20 years of age or older than 80 years of age, as well as during pregnant or lactating women, patients undergoing chemotherapy or hormone therapy, and those with a history or current diagnosis of cardiovascular or renal disease. Informed consent was obtained from each subject. This study was approved by the Institutional Review Board of Taichung Veterans General Hospital, Taiwan (CF13197) and registered at Clinical Trials.gov Identifier: NCT01964001.

The following data were recorded for all subjects before surgery: age, body weight, height, waist and hip circumference, smoking and drinking habits, exercise frequency, body mass index (BMI), and the waist/hip ratio were calculated. Dietary intake was assessed by dietitians, and 24 h diet recall was used after one month of the surgery. The dietary records were analyzed using the Nutritionist Professional software package (E-Kitchen Business Corp., Taichung, Taiwan) and the nutrient database was based on the Taiwan food composition table (Food and Drug Administration, Ministry of Health and Welfare, Taipei, Taiwan).

### 2.2. Blood Collection and Biochemical Measurement

Fasting blood specimens were collected in vacutainer tubes without anticoagulant (Becton Dickinson, Rutherford, NJ, USA) before and month after surgery. Serum and plasma were prepared after centrifugation (3000 rpm, 4 °C, 15 min) and were then stored at −80 °C until analysis. Hematological entities, such as blood urea nitrogen, creatinine, glutamic oxaloacetic transaminase, glutamic pyruvate transaminase (GPT), and lipid profiles were measured using an automated biochemical analyzer (Hitachi-7180E, Tokyo, Japan). The level of high sensitivity C-reactive protein (hs-CRP) was quantified by particle-enhanced immunonephelometry with an image analyzer (Dade Behring, Chicago, IL, USA). Plasma tumor necrosis factor-α (TNF-α) (R & D Systems Inc., Minneapolis, MN, USA) and interleukin-6 (IL-6) (eBioscience, San Diego, CA, USA) levels were measured using an enzyme-linked immunosorbent assay (ELISA) with commercially available kits, according to the manufacturer’s instructions.

Plasma coenzyme Q10 and vitamin E levels were measured using high-performance liquid chromatography (HPLC) and were detected with a UV detector at 275 nm and 292 nm, respectively [17,18]. Plasma malondialdehyde (MDA) was determined using the TBARs (thiobarbituric acid reactive substances) method, as described by Botsoglou [19]. The red blood cell (RBC) samples were washed with normal saline after removing the plasma. Then, the RBC samples were diluted with a 25x sodium phosphate buffer for superoxide dismutase (SOD) and glutathione peroxidase (GPx) measurements, with a 250x sodium phosphate buffer for the catalase (CAT) measurement. The antioxidant enzymes activities (CAT, SOD, and GPx) were determined in the fresh samples, and the methods used to measure these activities have been previously described [20,21,22]. The protein content of the plasma and RBC was determined based on the biuret reaction of the bicinchoninic acid (BCA) kit (Thermo, Rockford, IL, USA). The values of the antioxidant enzymes activities were expressed as unit/mg of protein. All analyses were performed in duplicate.

### 2.3. Statistical Analysis

The data were expressed as means and standard deviations (SD), as well as medians. A Kolmogorov–Smirnov test was used to examine the normal distribution of variables. A paired *t*-test was used to compare mean values for continuous variables before and after surgery. Pearson product moment correlation was used to examine the correlations between the levels of antioxidant capacity, oxidative stress, and inflammatory markers in HCC patients and the change of coenzyme Q10, antioxidant capacity, oxidative stress, and inflammation in HCC patients after surgery. Simple linear regression was used to examine the correlations between the levels of coenzyme Q10 and vitamin E, and antioxidant enzyme activity (GPx) in HCC patients. Statistical significance was set at *p* < 0.05. All statistical analyses were performed using SigmaPlot software (version 12.0, Systat, San Jose, CA, USA).

## 3. Results

### 3.1. Participant Characteristics

The characteristics and dietary intake of the subjects are shown in Table 1. In total, 70% of the subjects were males, and the mean age of the subjects were 59 ± 11 years old. The frequencies of smoking, drinking, and exercise habits were 17%, 9%, and 44%, respectively. Additionally, 39% of the subjects had been infected with hepatitis B, 17% of the subjects had been infected with hepatitis C, and 14% of the subjects were cirrhosis. More than half of the subjects (56%) had HCC recurrence. With regard to the hematological data, the subjects had significantly higher levels of blood urea nitrogen (BUN), creatinine, and high density lipoprotein-cholesterol (HDL-C) and lower levels of glutamic oxalocetic transaminase (GOT) and total cholesterol to high density lipoprotein-cholesterol ratios (TC-to-HDL-C ratios) (*p* < 0.01) after surgery. With regard to dietary intake, subjects had a significantly higher protein intake of total calories (*p* = 0.04) and lower fat intake (*p* = 0.07) after surgery.

### 3.2. Levels of Coenzyme Q10, Oxidative Stress, and Inflammation

The levels of coenzyme Q10, vitamin E, oxidative stress and inflammatory markers after surgery are shown in Table 2. After surgery, the subjects had a significantly lower levels of coenzyme Q10 (*p* = 0.01) and MDA (*p* < 0.01), and significantly higher levels of vitamin E (*p* < 0.01) and antioxidant enzymes activities (SOD, *p* < 0.01; CAT, *p* < 0.01; GPx, *p* = 0.04). With regard to inflammatory markers, the subjects had significantly higher levels of hs-CRP (*p* = 0.04), TNF-α (*p* < 0.01) and IL-6 (*p* < 0.01) after surgery.

### 3.3. Correlations between Coenzyme Q10, Oxidative Stress, and Inflammation 

The correlations between coenzyme Q10, oxidative stress, and inflammation in HCC patients are shown in Table 3. There was a significantly negative correlation between oxidative stress (MDA) and antioxidant enzymes activities (SOD, *p* = 0.05; CAT, *p* < 0.05, and GPx, *p* = 0.04) (Table 3). With regard to the correlation between antioxidant capacity and inflammation, CAT activity shown to be significantly negatively correlated with the level of hs-CRP (*p* = 0.02), and GPx activity was significantly negatively correlated with the levels of TNF-α (*p* = 0.02) and IL-6 (*p* < 0.01). 

Furthermore, we assessed the correlations between the changes in coenzyme Q10, oxidative stress, and inflammation in HCC patients, and these results are shown in Table 4. There was a significantly negative correlation between changes in the levels of coenzyme Q10 and inflammation markers (hs-CRP, *p* = 0.02; IL-6, *p* = 0.05) after surgery. In addition, changes in GPx activity were significantly negatively correlated with changes in oxidative stress (MDA, *p* = 0.06) and inflammation markers (hs-CRP, *p* < 0.05; IL-6, *p* = 0.04), and changes in SOD activity was significantly negatively correlated with changes in hs-CRP (*p* = 0.04) after surgery.

### 3.4. Correlations between Coenzyme Q10 and Antioxidant Capacity

**Figure 1:**
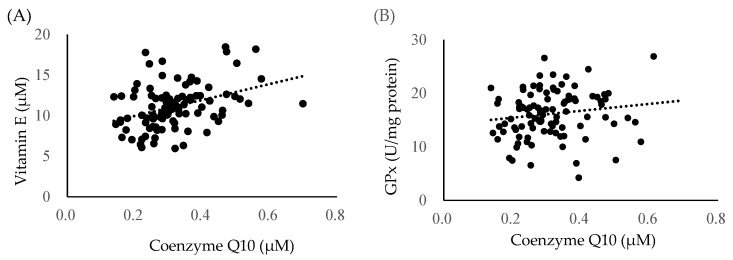
Correlations between the levels of coenzyme Q10 and vitamin E, and glutathione peroxidase activity (GPx) in HCC patients. (**A**) Correlation between the levels of coenzyme Q10 and vitamin E (β = 9.85, *p* < 0.01); (**B**) Correlation between the levels of coenzyme Q10 and glutathione peroxidase (GPx, β = 6.43, *p* = 0.04).

The correlations between coenzyme Q10 and antioxidant capacity is shown in Figure 1. The level of coenzyme Q10 was significantly positive correlated with vitamin E (β = 9.85, *p* < 0.01) and GPx activity (β = 6.43, *p* = 0.04) in HCC patients.

## 4. Discussion

This study is the first clinical study to investigate the coenzyme Q10 status in patients with HCC before and after surgery. The reference values of the level of coenzyme Q10 were suggested to be between 0.5 to 1.7 μM [23]. It is worth noting that the level of coenzyme Q10 was lower in patients with HCC before surgery (Table 2), and the level of coenzyme Q10 was further reduced significantly after surgery (Table 2, 0.34 ± 0.11 μM reduced to 0.30 ± 0.11 μM, *p* < 0.01). Coenzyme Q10 is a crucial cellular antioxidant [9,10], and our previous studies have demonstrated that coenzyme Q10 can significantly reduce oxidative stress and inflammation status via its antioxidant capacity [24,25,26]. In the present study, we found that the level of coenzyme Q10 was significantly correlated with vitamin E and antioxidant enzyme (GPx) activity in HCC patients (Figure 1). Patients with HCC suffer from a higher level of oxidative stress and inflammation [27], and a deficiency in coenzyme Q10 was found in HCC patients, we suggest that the administration of coenzyme Q10 supplements in patients with HCC during the surgery may be beneficial.

Surgical resection is the most efficient treatment of patients with HCC [3]. Over the past 10 years, surgical treatment has been considered safe, with an acceptable overall mortality rate (<5%), and resulted in good long-term survival (>50%) in HCC patients [28,29,30]. However, surgery may elicit the activation of systemic inflammatory, endocrine/metabolic, and immunological systems, which is referred to as the surgical stress response [4]. In the present study, it is interesting to note that, after one month of the surgery, patients with HCC exhibited significantly lower oxidative stress and higher antioxidant enzymes activities (Table 2). The level of oxidative stress (MDA) was significantly reduced by 15%, and the antioxidant activities of SOD, CAT, and GPx were significantly increased by 8.6%, 9.6%, and 3.1%, respectively. Although surgery improved the antioxidant capacity in HCC patients, compared with the administration of antioxidants, these increases were not high [24,25,26,31]. Our previous clinical intervention study have observed that the administration of coenzyme Q10 at a dose of 300 mg/day in patients with HCC could significantly increase the antioxidant enzymes activities of SOD by 67.3%, CAT by 42.6%, and GPx by 26.5% and reduce oxidative stress by 17.3% after 12 weeks of supplementation [31]. In the present study, we found a non-statistically negative correlation between the changes in coenzyme Q10 and the changes in oxidative stress (MDA) after surgery (Table 4) and the level of coenzyme Q10 was significantly positive correlated with vitamin E and antioxidant enzyme (GPx) activity in HCC patients (Figure 1). Additionally, the latest clinical study conducted by Cannistrà et al. [32] demonstrated that the rates for the morbidity, muscle weakness, and pleural effusion were significantly lower in elderly patients with HCC during surgical resection after coenzyme Q10 supplementation. Since hepatocarcinogenesis is associated with severe oxidative stress [33], antioxidants, such as coenzyme Q10, could be considered a complementary treatment strategy for patients with HCC after surgery.

With regard to inflammation status, surgery may elicit systemic immunological and inflammatory responses, including the activation of polymorphonuclear leucocytes, macrophages, monocytes, platelets, and mast cells, as well as the hyper-production of cytokines (e.g., IL-6 and TNF-α) [4]. Similar findings were observed in the present study, patients with HCC had a significantly higher inflammation status after surgery (Table 2). It is not surprising that a higher inflammation status in these patients due to the response to the surgery. However, in the present study, we found that changes in coenzyme Q10 were significantly negatively correlated with inflammation after surgery (Table 4). Most patients with HCC occur in cirrhotic livers and are associated with oxidative stress [30]. It is already known that inflammation is associated with NF-kB activation [34], and NF-κB can be activated by ROS, subsequently resulting in the upregulation of the expression of pro-inflammatory cytokines. Oxidative stress can also induce the production of a variety of cytokines in Kupffer cells, thus increasing inflammation and the apoptosis of hepatic cells [35]. As inflammation is correlated with oxidative stress, using antioxidants to inhibit the inflammatory-activating cascade could be considered. We suggest that administering antioxidants, such as coenzyme Q10, in HCC patients, particularly in those with a lower coenzyme Q10 status after surgery, is worth attempting.

## 5. Conclusions

In this clinical study, we clarified that patients with HCC exhibited lower levels of coenzyme Q10 and oxidative stress after surgery, and the level of coenzyme Q10 was associated with antioxidant capacity and inflammation. As hepatocarcinogenesis is chronic inflammation associated with severe oxidative stress, antioxidants, such as coenzyme Q10, could be considered a complementary treatment strategy for patients with HCC, particularly those with higher inflammation after surgery. 

## Figures and Tables

**Table 1:** Characteristics and dietary intake of subjects ^1^.

Subjects Characteristics			
males (*n*, %)	50 (70%)		
age (years)	59.2 ± 11.3 (59.0)		
SBP (mmHg)	125.3 ± 11.3 (123.0)		
DBP (mmHg)	76.9 ± 12.3 (76.5)		
waist circumference (cm)	88.9 ± 8.6 (90.0)		
waist hip ratio	0.9 ± 0.1 (0.9)		
BMI (kg/m^2^)	24.4 ± 5.8 (24.0)		
current smokers ^2^ (*n*, %)	12 (17%)		
drink alcohol ^3^ (*n*, %)	6 (9%)		
exercise ^4^ (*n*, %)	31 (44%)		
Hepatitis B, *n* (%)	28 (39%)		
Hepatitis C, *n* (%)	12 (17%)		
Cirrhosis, *n* (%)	10 (14%)		
Recurrence, *n* (%)	40 (56%)		
**Hematology**	**Before Surgery**	**After Surgery**	***p* Values**
BUN (mmol/L)	9.9 ± 3.6 (9.3)	11.6 ± 4.8 (10.7)	<0.01
creatinine (μmol/L)	70.7 ± 17.7 (70.7)	79.6± 26.5 (70.7)	<0.01
GOT (IU/L)	63.2 ± 61.4 (38.0)	40.1 ± 36.3 (40.0)	<0.01
GPT (IU/L)	60.4 ± 57.4 (38.5)	54.3 ± 47.8 (40.0)	0.79
TC (mmol/L)	4.3 ± 1.0 (4.2)	4.3 ± 0.8 (4.2)	0.20
TG (mmol/L)	1.1 ± 0.5 (1.0)	1.1 ± 0.4 (1.0)	0.64
LDL-C (mmol/L)	2.8 ± 0.9 (2.8)	2.7 ± 0.8 (2.6)	0.97
HDL-C (mmol/L)	1.2 ± 0.3 (1.1)	1.3 ± 0.3 (1.2)	<0.01
TC/HDL-C	3.8 ± 1.2 (3.5)	3.5 ± 1.0 (3.4)	<0.01
**Dietary Intake**			
energy (kcal/day)	1952.0 ± 546.8 (1819.9)	1796.2 ± 405.9 (1793.3)	0.21
protein (g/day)	67.6 ± 23.3 (64.9)	68.7 ± 20.6 (72.6)	0.28
% of total calories	13.7 ± 3.2 (13.0)	15.2 ± 3.7 (14.9)	0.04
fat (g/day)	66.7 ± 32.6 (54.8)	55.4 ± 22.0 (53.1)	0.07
% of total calories	30.3 ± 10.1 (29.0)	26.8 ± 7.7 (26.4)	0.10
carbohydrate (g/day)	274.0 ± 92.9 (253.0)	264.3 ± 66.9 (258.9)	0.52
% of total calories	55.9 ± 10.8 (57.0)	58.3 ± 7.8 (59.4)	0.26
dietary fiber (g/day)	14.5 ± 7.3 (13.0)	14.7 ± 7.4 (12.7)	0.47
cholesterol (mg/day)	244.1 ± 178.0 (186.6)	233.3 ± 147.3 (225.7)	0.63
vitamin E (mg α-TE/day)	271.8 ± 489.1 (13.0)	563.8 ± 888.1 (18.0)	0.28

^1^ mean ± SD (medians); ^2^ current smokers: individuals who current smoke one or more cigarettes per day; ^3^ drink alcohol: individuals who regularly drink one or more drink per day; ^4^ exercise: individuals who exercise at least three times every week. BMI: body mass index; BUN: blood urea nitrogen; DBP: diastolic blood pressure; GOT: glutamic oxaloacetic transaminase; GPT: glutamic pyruvate transaminase; HDL-C: high-density lipoprotein-cholesterol; LDL-C: low density lipoprotein-cholesterol; SBP: systolic blood pressure; TC: total cholesterol; TG: triglyceride.

**Table 2:** Levels of coenzyme Q10, oxidative stress, and inflammation ^1^.

	Before Surgery	After Surgery	*p* Values
Coenzyme Q10 (µM)	0.34 ± 0.11 (0.32)	0.33 ± 0.11 (0.28)	0.01
Vitamin E (µM)	10.4 ± 2.9 (11.8)	11.8 ± 2.6 (10.2)	<0.01
Oxidative Stress			
MDA (µM)	1.68 ± 0.40 (1.60)	1.43 ± 0.43 (1.36)	<0.01
Antioxidant Enzymes Activity			
SOD (U/mg protein)	13.2 ± 6.4 (12.4)	15.3 ± 6.8 (13.5)	<0.01
CAT (U/mg protein)	13.2 ± 5.7 (11.4)	15.3 ± 7.2 (12.5)	<0.01
GPx (U/mg protein)	15.7 ± 4.9 (16.3)	16.4 ± 4.6 (16.8)	0.04
Inflammatory Markers			
hs-CRP (mg/L)	4.6 ± 8.5 (1.3)	4.8 ± 5.0 (2.5)	0.04
TNF-α (pg/mL)	0.4 ± 0.7 (0.1)	0.9 ± 1.0 (0.8)	<0.01
IL-6 (pg/mL)	2.3 ± 1.9 (1.6)	3.6 ± 2.8 (2.5)	<0.01

^1^ mean ± SD (medians). CAT: Catalase activity; MDA: Malondialdehyde; GPx: glutathione peroxidase; HCC: hepatocellular carcinoma; hs-CRP: high sensitivity C-reactive protein; IL-6: interleukin-6; SOD: superoxide dismutase; TNF-α: tumor necrosis factor-α.

**Table 3:** Correlations^1^ between coenzyme Q10, oxidative stress, and inflammation in HCC patients.

	Oxidative Stress	Inflammatory Markers		
	MDA (μM)	hs-CRP (mg/dL)	TNF-α (pg/mL)	IL-6 (pg/mL)
Coenzyme Q10 (μM)	0.13	0.14	−0.06	0.02
vitamin E (μM)	0.07	0.09	−0.04	0.24
SOD (U/mg protein)	−0.10 ^†^	0.00	0.18	0.13
CAT (U/mg protein)	−0.10 *	−0.15 *	0.51	0.14
GPx (U/mg protein)	−0.11 *	−0.08	−0.17 *	−0.19 *

^1^ correlation coefficient (*r*). * *p* < 0.05; ^†^ *p* = 0.05. CAT: Catalase activity; MDA: Malondialdehyde; GPx: glutathione peroxidase; HCC: hepatocellular carcinoma; hs-CRP: high sensitivity C-reactive protein; IL-6: interleukin-6; SOD: superoxide dismutase; TNF-α: tumor necrosis factor-α.

**Table 4:** Correlations^1^ between changes in coenzyme Q10, oxidative stress, and inflammation.

	Changes of Oxidative Stress	Changes of Inflammatory Markers		
	MDA (μM)	hs-CRP (mg/dL)	TNF-α (pg/mL)	IL-6 (pg/mL)
Coenzyme Q10 (μM)	−0.11	−0.23 *	0.18	−0.16 *
vitamin E (μM)	0.01	0.09	−0.13	0.08
SOD (U/mg protein)	−0.04	−0.16 *	0.04	−0.07
CAT (U/mg protein)	−0.09	−0.03	0.24	0.01
GPx (U/mg protein)	−0.18 ^†^	−0.26 *	−0.10	−0.20

^1^ correlation coefficient (*r*). * *p* < 0.05; ^†^ *p* = 0.06. CAT: Catalase activity; MDA: Malondialdehyde; GPx: glutathione peroxidase; HCC: hepatocellular carcinoma; hs-CRP: high sensitivity C-reactive protein; IL-6: interleukin-6; SOD: superoxide dismutase; TNF-α: tumor necrosis factor-α.

## Abbreviations

The following abbreviations are used in this manuscript:

BMI	body mass index
BUN	blood urea nitrogen
CAT	catalase
DBP	diastolic blood pressure
GPx	glutathione peroxidase
GOT	glutamic oxaloacetic transaminase
GPT	glutamic pyruvate transaminase
HDL-C	high-density lipoprotein-cholesterol
hs-CRP	high sensitivity C-reactive protein
IL-6	interleukin-6
LDL-C	low density lipoprotein-cholesterol
MDA	malondialdehyde
SBP	systolic blood pressure
SOD	superoxide dismutase
TC	total cholesterol
TG	triglyceride
